# Supplementary material for: Are carbon emissions trading and green financial instruments synergistic? -Comprehensive quantitative research based on content analysis
Source: PLoS One. 2024 Mar 7;19(3):e0298601. doi: 10.1371/journal.pone.0298601 (PMC10919601; doi:10.1371/journal.pone.0298601)
Supplement: S1 Data — (ZIP) [file pone.0298601.s001.zip › Data/地级市Z_F工作报告.zip!/╕╩╦α/╝╬╙°╣╪╩╨/└┤╫╘╣1⁄2╓┌║┼í╛┬φ┐╦╩2╛▌═°í┐_2019.docx]

各位代表：

现在，我代表市人民政府向大会作报告，请予审议，并请各位政协委员和其他列席人员提出意见。

一、2018年工作回顾

过去一年，是我国改革开放40周年、酒钢建厂60周年，也是全市上下凝心聚力、攻坚克难、真抓实干的一年。一年来，面对复杂严峻的外部环境和艰巨繁重的改革发展任务，在省委、省政府和市委的坚强领导，市人大和市政协的监督支持下，我们全面贯彻习近平新时代中国特色社会主义思想和党的十九大精神，深入落实习近平总书记视察甘肃重要讲话和“八个着力”重要指示精神，坚持稳中求进工作总基调，践行新发展理念，按照高质量发展要求，统筹推进稳增长、调结构、促改革、惠民生、防风险各项工作。初步预计，全年完成地区生产总值288亿元，同比增长6%；城乡居民人均可支配收入39045元和19219元，分别增长7%、8%；一般公共预算收入19.68亿元，增长7.2%。全市经济总体呈趋稳向好态势。

——项目建设务实推进。扎实开展“项目建设提质年”活动，举办项目管理、项目融资、PPP项目等培训班，实施项目的科学性和有效性不断提高。立足大局谋划增量项目，聚焦全面建成小康短板、乡村振兴、十大绿色生态产业谋划项目222个，总投资规模逾1100亿元。其中：34个总投资415亿元的项目列入全省绿色生态产业发展规划；开工建设102个，当年完成投资53亿元；开展前期工作50个，总投资262亿元。务实创新优化存量项目，对56个谋划层次不高、论证不充分、缺乏投资吸引力的项目，重新“回炉”或淘汰。落实领导包抓项目责任制，抓好政府投资、重点前期、市级领导包抓项目“三个清单”，110个市列重点项目开工率81.8%，49个领导包抓重大项目完成投资41.3亿元。争取国拨省补投资项目28个、资金3.55亿元，开工率92.9%、完成投资5.03亿元。全年固定资产投资降幅逐季收窄。

——工业转型初见成效。制定促转型系列政策措施和实体经济奖励办法，推进工业经济高质量发展。全力支持酒钢转型升级，牙买加165万吨氧化铝项目成功复产，天成彩铝绿色短流程铸轧铝项目基本建成，宏汇能源一期150万吨煤炭分质利用项目投料试生产。军民融合发展起步良好，与中核集团达成研发及生活基地建设意向，空军转运站前期工作有序开展。地方工业转型取得实质性突破，一特多功能专用汽车生产线建成投产，筑友装配式建筑智能制造基地顺利实施。完成新型智慧城市建设顶层设计，鑫海成大数据中心建设积极推进。工业园区低碳园区试点基本完成，循环化改造加快推进，省级高新技术产业开发区获批。完成规上工业增加值161.7亿元，增长3.3%；规上企业实现盈利8.3亿元。

——文旅产业快速发展。制定旅游强市、乡村旅游、智慧旅游等政策措施，修订全域旅游扶持奖励办法，推进景城、产城、城乡一体化发展。关城大景区基本建成，世界文化遗产公园初具规模，丝绸之路文化博览园即将竣工，峪泉古街稳步推进，关城旅游专线、景区连接线开通投运。水立方文化旅游乐园建成运营。加大旅游宣传营销力度，培育6条旅游新航线。“敦煌行·丝绸之路国际旅游节”开幕式、嘉峪关国际短片电影展、国际房车博览会等成功举办。坚持“大旅游”“小旅游”一起抓，全域旅游规划完成评审，累计建成74家星级农家乐、40家乡村旅馆，6个乡村旅游示范点建设进展顺利。文旅创意产品研发力度加大，获得全国特色旅游商品大赛银奖和甘肃旅游商品大赛金奖各1项。旅游接待人数突破1000万大关，收入突破70亿元，分别增长22%和29%；带动文化及相关产业增长13.5%。完成第三产业增加值109亿元，增长9.7%。

——乡村振兴起步良好。深化农村集体产权制度和“三变”改革，整合支农资金500多万元，撬动社会资本7000余万元，发展农民股东998户，完成3个试点村清产核资、入股分红等工作。土地确权颁证率99%，耕地流转率31.9%。加快发展戈壁农业，推进镜铁区农业示范园、酒钢宏丰和新城镇戈壁农业产业园建设，开工建设日光温室268座，建成108座。培育家庭农场、农业龙头企业等新型经营主体13家，农民专业合作社达到303个。扎实开展“双促双增”行动，建立市级领导联系、部门包村、干部职工精准对接机制，实现17个行政村农业提质增效、6071户农村居民持续增收政策、措施、行动全覆盖。启动农村人居环境整治三年行动，对各村组居民院落及周边环境全面清理整治，累计建成10个省级“千村美丽”示范村，农村环境面貌明显改观。

——城市功能更加完善。强化规划引领，完成三镇规划编制，实现全市集中建设区控规全覆盖。加快补齐交通基础设施短板，G312嘉峪关至清泉公路、驻地部队道路建设等项目有序推进，酒嘉绕城高速前期工作进展顺利。五一路、朝阳路、建设路等7条老旧道路改造和南市区7条道路扩建全面完工，城区道路提升改造工程基本完成。新建改造地下管网190公里。启动新三年棚户区改造工程，征迁改造房屋1062套，扩建、翻建老旧小区住房12750套。推进“厕所革命”，新建城市、农村和旅游景点公厕35座。实施公共服务领域市场化改革，深入开展全域全城无垃圾创建，持续深化出租车、景区景点、马路市场等专项整治，启动“拆违建、排隐患、保安全”整治行动，全国文明城市创建成果持续巩固。

——改革开放逐步深化。深入开展“放管服”改革突破年活动，梳理确定依申请类政务服务事项993项，精简取消证明104项，公布“最多跑一次”790项，下沉社区受理便民服务72项。创建“政府服务中心+分中心+社区服务中心”政务服务模式，初步实现服务群众事项“就近办”，办事流程简化11.8%、要件压减30.2%、承办时间缩减32%。推进“互联网+政务服务”一体化平台建设，完善并联审批及项目全程代办制，依申请类政务服务事项网上办理达到99.3%、开办企业注册时间压缩至3个工作日、不动产登记5个工作日。全面推行证照分离、先照后证改革，办理“多证合一”营业执照1.1万余份，“放管服”改革模式在全省作经验交流。深化国资国企改革，制定“1+N”配套文件，28家国有、集体企业和自收自支事业单位整合为9家有限责任公司，国有资本经营收益1748万元，增长1倍以上。推进企业办社会职能剥离，7家企业“三供一业”分离移交基本完成。开放步伐不断加快，编制完成枢纽经济、物流业发展等专项规划，酒钢多式联运物流园、国际港务区空港物流园等项目有序推进。与西班牙纳瓦拉自治区潘普洛纳市缔结为友好城市，开展文化旅游、教育卫生等领域交流合作。加大精准招商力度，组织参加深圳文博会、兰洽会等展会，引资到位152.87亿元；预计全年外贸进出口总额20.2亿元，增长13%。

——营商环境不断优化。落实市级领导联系非公企业制度，制定促进民营经济健康发展意见“30条”，修订非公有制经济奖励办法，召开地企联席会、政银企对接会、民营企业家座谈会，兑现实体经济暨非公有制经济奖励扶持资金1317万元。积极开展“零收费”试点，落实减税降费政策，全年减免税6.69亿元、退税1.8亿元。引导金融机构优化信贷结构，创新金融产品，缓解融资难题，全市各项贷款余额532.4亿元，同比增长2.5%；其中小微企业贷款余额96.03亿元，增长25.8%。办理企业不动产抵押登记262件，主债权金额21.36亿元，企业获得感和发展信心不断增强。

——生态环保力度加大。践行“绿水青山就是金山银山”理念，完成义务植树5.2万株，退耕还林1699亩，城市建成区新增绿地1784亩，绿地率、绿化覆盖率达到38.9%和40%。草湖国家湿地公园建设、讨赖河生态环境治理扎实推进。集中解决环境突出问题，中央督察反馈18项问题整改完毕；省级环保督察反馈问题完成整改7项，正在整改24项。持续开展大气、水、土壤污染防治，完成农村冬季清洁取暖改造1582户，餐饮场所清洁能源使用率100%，餐厨垃圾实现规范处理和封闭运行。酒钢集团投资6.24亿元的燃煤火电机组超低排放改造工程、黑山矿区综合整治全面完成，民丰化工土壤污染修复、嘉北污水处理厂建设稳步推进。河长制、湖长制全面推行。集中式饮用水水源地保护专项行动有序开展。空气质量优良天数304天，优良率83.3%。单位生产总值能耗下降4.58%。

——创新能力逐步增强。持续加大科技创新投入，全社会研发经费（R&D）支出占地区生产总值比重4.9%、综合科技进步水平指数达到52%、每万人发明专利拥有量7.17件，均居全省前列。组织实施各类科技计划41项，其中列入省级项目13项。新增省级知识产权优势企业、科技创新型企业和企业技术中心等15家，列入国家和省级科技型中小企业23家，酒钢天成彩铝入选全省战略性新兴骨干企业，甘肃钢铁职业技术学院成功创建首家“省市共建甘肃省冶金技术重点实验室”。积极落实“双创”扶持政策，建成国家和省级创新创业服务平台、孵化示范基地10个，入孵企业311家，吸纳带动就业2370余人。

——民生福祉持续增进。完成省政府为民办实事涉及我市6件，配置高铁南站电梯、开通镇村公交车、关城景区向市民永久免费开放等8件市政府为民办实事全面完成。优先保障和改善民生，财政支出的80.2%投向民生领域，增长5.5%。持续加大教育基础设施建设，新建、改扩建项目12个，胜利路小学投入使用，福民街、建林街幼儿园建成，实验中学教学楼改扩建项目全面开工。不断拓宽就业渠道，组织技能培训3125人，劳务输转7252人，新增就业8381人，高校毕业生就业率93%，城镇登记失业率2.8%。农民工工资清欠率93.5%。健全社会保障体系，推进社会保险扩面征缴，启动机关事业单位养老保险征缴工作。全面推进健康嘉峪关建设，建立现代医院管理制度，完善分级诊疗机制，扩大异地就医直接结算范围；中医院综合楼、酒钢医院综合门诊楼改扩建项目主体建成。康乐寿医护养老中心建成投用，全国第三批居家和社区养老服务改革试点工作全面启动。国家食品安全示范城市通过省级验收。严格落实安全生产责任制，未发生较大安全责任事故。深入开展“扫黑除恶”专项斗争行动，加强信访维稳工作，有效化解矛盾纠纷，推进互联网金融、非法集资等专项治理，社会大局保持和谐稳定。扎实开展双拥共建活动，军地军民关系更加融洽。

——自身建设明显加强。坚持把政治建设贯穿政府工作全过程，牢固树立“四个意识”，坚定“四个自信”，践行“两个维护”，推进政府系统党的十八大以来各类巡视、督察、审计等问题整改，确保中央大政方针及省委、省政府和市委决策部署落实到位。扎实开展“转变作风改善发展环境建设年”“解放思想大讨论”“能力素质大提升”“行政效能大提速”等活动，组织开展审计监督、小康指标等专题培训18期，培训干部1.1万余人次。严格执行中央八项规定及实施细则精神，厉行勤俭节约，一般性支出压减8%，“三公”经费支出不断下降。完善政府投资项目造价审核、公共资源交易、审计监督等制度，节约财政资金1.7亿元。制定落实省政府“十不准”负面清单，政府部门办事效率和服务效能明显提高。深入推进法治政府建设。成立市政府专家决策咨询委员会。主动接受各方面监督。办理人大代表建议224件、政协委员提案和建议105件，办复率100%、满意率98%。全面落实党风廉政建设主体责任和“一岗双责”，加大逐级廉政约谈频次，层层传导责任压力，政府系统党风廉政建设和反腐败斗争持续加强。

同时，统计调查、应急减灾、人防气象、粮食供销、外事侨务、市志档案、工青妇幼等工作扎实推进，妇女儿童、老年人、残疾人权益得到有效保障。

各位代表，过去一年，全市上下团结一心、砥砺前行，推动高质量发展迈出坚实步伐，取得这样的成绩实属不易。这是省委、省政府和市委正确领导的结果，是市人大、市政协、嘉峪关军分区、酒钢集团公司、中核四○四、中核龙瑞公司及驻嘉各单位鼎力支持的结果，是全市各族人民团结拼搏的结果。在此，我代表市人民政府，向全市各族人民、人大代表、政协委员，向各民主党派、工商联、无党派人士、人民团体和社会各界人士，向驻嘉部队、武警官兵、政法干警和民兵预备役人员，向所有关心支持嘉峪关发展的朋友们，表示衷心的感谢并致以崇高的敬意！

各位代表，在总结成绩的同时，我们更加清醒地认识到，全市经济社会发展还存在诸多困难和问题。一是经济下行压力依然较大，新旧动能接续转换较慢，经济总量不大与发展质量不优问题并存，民间投资意愿低迷，部分经济指标未达到预期目标，经济企稳基础不牢；二是基础设施领域短板突出，交通水利、产业发展、生态环境、社会民生和城市基础设施欠账较多，还有很多薄弱环节，严重制约着经济转型升级和城市转型发展；三是全面建成小康任务艰巨，补齐科技进步贡献率、文化及相关产业占比、公共交通服务指数、工业固体废物综合利用率、单位GDP能耗、森林覆盖率等短板难度较大；四是改革开放创新动力不足，“放管服”改革还不到位，对外开放层次不高，营商环境有待优化，创新资源严重短缺，构建与高质量发展相适应的体制机制还需久久为功；五是防范化解风险能力有待提高，传统支柱产业市场竞争力减弱，刚性支出增长较快，财政收支矛盾突出，偿债压力不断加大；六是工作作风需要持续改进，政府个别部门和干部责任担当、服务意识不强，推拖绕、打折扣、中梗阻等现象依然存在，攻克发展难题的能力不强、韧劲不足。对于这些问题，我们要以“自我革命”的勇气、坚韧不拔的毅力、务实有效的措施，认真加以解决。

二、2019年主要目标和重点任务

2019年是新中国成立70周年，是决胜全面建成小康社会关键之年，我们面临的外部环境更加严峻，推进改革发展稳定的任务更加艰巨。做好今年各项工作，责任重大，意义特殊。政府工作的总体要求是：以习近平新时代中国特色社会主义思想为指导，全面贯彻党的十九大和十九届二中、三中全会精神，统筹推进“五位一体”总体布局，协调推进“四个全面”战略布局，深入落实习近平总书记视察甘肃重要讲话和“八个着力”重要指示精神，认真贯彻“五坚持”大政方针，扎实做好“六稳”工作，突出抓好十大绿色生态产业，统筹推进稳增长、促改革、调结构、惠民生、防风险工作，确保经济持续平稳发展和社会和谐稳定，为建成高水平小康社会打下决定性基础。

综合考虑发展基础和条件，本着实事求是、量力而行、尽力而为的原则，经济社会发展主要预期目标是：地区生产总值增长6%，固定资产投资增长6%左右，全社会消费品零售总额增长8%左右，一般公共预算收入增长6%，城乡居民人均可支配收入分别增长6%、7%，居民消费价格指数涨幅控制在3%以内，单位生产总值能耗和主要污染物排放完成省政府下达的控制指标。

要实现上述目标，我们必须以高质量发展为引领，以供给侧结构性改革为主线，在落实中央“巩固、增强、提升、畅通”八字方针上下功夫，补齐发展短板抓项目，做强工业经济抓转型，整合文旅资源抓提升，发展通道物流抓突破，推动乡村振兴抓示范，强化城市管理抓提质，推进改革开放抓深化，优化营商环境抓服务，坚持创新驱动抓引领，保障改善民生抓提高，全力做好以下重点工作：

（一）抓实项目促进投资，夯实基础补齐短板。开展“项目建设攻坚年”活动，准确把握政策导向，以高质量发展引领项目建设，全面提高项目质量效益。

精准谋划争取项目。抢抓国家实施新一轮西部大开发战略机遇，聚焦交通能源、生态环保、城市建设、农业农村、社会民生等关键领域和薄弱环节，重点围绕“一带一路”建设总体布局和全省构建十大绿色生态产业体系，深入研究分析，加强项目储备，动态完善重大项目库，形成远近结合、梯次接续、良性循环格局。加强与国家部委、省直部门的汇报沟通和深度对接，力争更多项目纳入国家和全省计划盘子。

聚力攻坚重点项目。落实领导包抓项目责任制，坚持前期项目抓开工、新建项目抓进度、续建项目抓竣工、建成项目抓投产，推进实施176个、总投资591亿元的重点项目，当年计划投资121亿元。优先实施宏晟电热超低排放改造、东兴铝业电解槽优化、戈壁设施农业示范园、草湖国家湿地公园、国际空港物流园、鑫海成大数据中心、中核甘肃核技术产业园生活基地、天成彩铝铝材精深加工、河湖连通综合利用、酒嘉绕城高速等50个带动性强的十大绿色生态产业项目。

着力提高招商实效。把招商引资作为增投资、促转型的“一号工程”，修订招商引资优惠政策，完善地企联动机制，压实招商引资责任。筛选一批发挥产业优势、凸显资源禀赋、投资强度大、市场前景好的项目，开展精准招商、驻点招商、以商招商。抢抓发达地区产业重新布局“窗口期”，紧盯文化旅游、物流枢纽、生态环保等领域，主动加强与骨干龙头企业上下游合作伙伴的深度对接，切实提高项目履约率和资金到位率。

（二）加快转型升级步伐，做强做优工业经济。围绕国家深入实施中国制造2025纲要、支持老工业基地调整改造等契机，以制造业高端化引领转型，推进传统优势产业二次创业、新兴接续产业加速发展。

加快传统产业提质增效。重点围绕钢、铝优势产业建链延链补链强链，做大先进装备制造、新型有色金属等产业，提高工业经济质量和效益。坚定不移支持酒钢开展技术循环化改造和产品高端化升级，加快7号高炉改造复产，促进煤炭分质利用、粉矿悬浮磁化焙烧技改等项目投产达产。加快推进装配式建筑智能制造产业基地、不锈钢罩式楼及配套制氢站、高精度交通用铝板等项目建设。支持一特汽车重卡、皮卡、轻卡等车型生产达产，加大市场培育和开发力度。

培育发展新兴接续产业。持续加强与央企等优势大企业合作，推进重大项目建设。深化与中核四〇四、中核龙瑞、核工业北京地质研究院合作，加快推进研发中心、核装备制造等项目落地。培育发展低空旅游、航空运动、应急救援等通用航空产业。推动互联网、大数据、人工智能与制造业深度融合，加快建设工业云计算大数据平台，促进数字经济与实体经济融合发展。

有效盘活园区闲置资源。支持工业园区停产半停产企业采取兼并重组、老旧厂房改造等方式，盘活土地、厂房等闲置资产。依法稳妥处置“僵尸企业”，“一企一策”破除无效供给。创新用地供应方式，运用经济手段对有限土地重新配置。培育和发展标准厂房租赁市场，推进工业用地租赁、先租后让、租让结合和弹性年期出让供应，有效发挥园区的产业集聚和项目载体作用。

（三）整合特色资源要素，提升全域旅游品质。制定推动文化旅游产业高质量发展专项行动计划，以“文化+旅游+产业”发展新模式，培育天空、地面、地下立体旅游新产品，构建景城、产城、城乡一体化全域旅游新格局。

整合文化旅游资源。积极联合文化旅游品牌企业，共同建设以关城为核心，世界文化遗产公园、长城第一墩、戈壁大峡谷地质公园、石关峡景区及黑山岩画等为支撑点，历史文化、自然景观与工业文明融为一体的文化旅游园区，实现资源变资产、资产变资本、资本出效益。放大方特欢乐世界品牌效应，确保丝路博览园“五一”前开园，峪泉古街如期建成运营。构建“快旅慢游”交通体系，打通景区连接“最后一公里”，延长游客停留时间，促进旅游消费。坚持文旅商体深度融合，培育发展自驾、低空、户外、徒步、冰雪、运动体验等新型旅游业态，着力打造全国运动休闲城市和国际旅游目的地城市。

加强文化软实力建设。坚持保护与开发利用并重，修编《万里长城—嘉峪关文物保护规划》，实施人文古迹保护和历史再现工程。以丝路长城文化研究院为依托，成立文化创意产业促进中心，建设文化旅游创意产业园区，积极筹办全省大学生文化创意设计和西部创客设计大赛。加快建设“一馆两中心”。优化整合公益性文化资源，成立文化艺术交流中心，培育专业和民间演艺团队。按照4A级旅游景区标准和“一街一品”理念，打造具有文化底蕴、景观精致、特色鲜明、业态集中的主题街区、公园。巩固旅游标准化建设成果，提升旅游服务整体质量。加大旅游宣传推介力度，办好国际铁人三项赛、国际短片电影展、国际房车博览会、全国机器人运动大赛等节会赛事，全面提升城市吸引力和影响力。

推动乡村旅游扩面提质。培育发展以田园风光为韵、村落民宅为形、生态农业为基的乡村旅游，全面启动6个特色旅游示范点和田园综合体建设，积极开发农业观光、生态体验、休闲度假、康体养生等多业态旅游产品。探索建立“景区+企业+农户+市场”发展模式，引导鼓励农村居民以房屋、土地、资金、劳动力入股等多种形式参与旅游开发。完善乡村旅游服务标准，加大现有农家乐规范提质达标力度，推进乡村民宿特色化、品牌化发展。

（四）发挥交通区位优势，加快发展商贸物流。抓住国家将嘉峪关布局为区域级流通节点城市的契机，抢占“一带一路”建设通道制高点，着力打造综合物流枢纽城市、现代物流集散中心和区域性陆港经济区。

培育发展现代物流产业。以现代物流产业为先导推动经济转型升级，发展多式联运、区域分拨和物流配送，全面推进物流通道、节点、载体、网络建设。优化完善路网连接和配套设施，加快火车南站客运中心、嘉峪关火车站货运中心建设和铁路编组站扩能改造进度，推进客运与货运分离。大力发展陆港经济，谋划建设大型煤炭、大宗矿产品仓储、集散和交易中心，建设酒钢多式联运物流园、铁路物流基地，推进大宗货物运输“公”转“铁”，降低物流运输成本。加快国际港务区建设，实施嘉峪关机场改扩建、航空口岸、空港物流园等项目。积极争取保税物流中心（B型）。吸引国内外知名现代物流企业落户，拓展物流产业链，打造智慧物流平台。

推动商贸服务业提档升级。顺应城乡居民对美好生活的新需求，聚焦信息、绿色、住房、旅游休闲、教育文化体育、养老健康家政六大领域，培育网络、定制、体验、智能、时尚等消费新热点。聚力盘活商贸存量资源，加快建设嘉丰源大厦、紫轩国际、雄关商厦等城市综合体，弥补消费不足、人气不旺短板。落实“互联网+流通”行动计划，培育跨境电商企业，提升电商快递物流集散中心服务功能，引导商贸流通和服务型企业线上线下融合发展。改善消费环境，完善产品和服务全过程安全防范机制，创成国家食品安全示范城市，让老百姓吃的放心、穿的称心、用的舒心。

（五）抓好试点示范建设，推动乡村全面振兴。持续推进“双促双增”行动，以优化供给、提质增效、农民增收为目标，深化农业供给侧结构性改革，推进农业强起来、农村美起来、农民富起来。

做精特色优势产业。争取省政府绿色生态产业发展基金，利用荒滩、戈壁、盐碱地等闲置土地发展戈壁农业。突出“新、特、精、优”特点，做精绿色食品、反季果蔬、高效制种、优良畜禽等产业。以恒翔现代农业、野麻湾高新农业示范园等为依托，大力发展采摘、观光农业。提升农产品深加工水平，加快构建加工、冷链、仓储、物流、销售体系。培育新型职业农民，发掘壮大 “土专家”“田秀才”“农创客”等懂农业、爱农村、爱农民的人才队伍。

深化农村综合改革。全面推进农村集体产权制度和农村“三变”改革，盘活农村自然资源、存量资产和人力资本，壮大集体经济，增加农民收入。实施农民专业合作社能力提升工程，加大龙头企业引进和培育力度，鼓励创新模式，发展联合专业合作社，推行标准化管理，提高整体质量。加强和规范村务监督，健全村民共建共治共享体系。设立农村金融综合服务室。全面完成农业综合水价改革。

推进农村面貌持续改善。深入实施农村人居环境整治三年行动，打造美丽庭院、美丽村庄、美丽田园。继续推进农村垃圾、污水和村容村貌整治，建制村公厕覆盖率100%，农村卫生厕所普及率90%左右。实现农药化肥使用零增长，废旧农膜回收利用率80%以上，畜禽养殖废弃物、秸秆综合利用率70%和85%以上，建成全省农村人居环境整治示范工程。深化“大棚房”专项整治行动，全面清理占用耕地建设非农设施。

（六）强化生态环境保护，提升城市品质品位。围绕打造文明之城、康养之地、宜居之家，优化空间布局，完善服务功能，提高治理水平。

提升生态环境质量。推动园林绿化“提品质、增绿量、强管理”，开展景区、公园、居住区、城区周边防护林带等主要公共空间绿化美化改造。巩固扩大退耕还林成果，推进农村道路、村庄周围、房前屋后绿化。启动讨赖河安远沟至嘉酒分界段生态环境综合治理工程。持续打好蓝天、碧水、净土保卫战，巩固中央环保督察整改成效，做好省级环保督察反馈问题整改落实。抓好重点领域节能减排、循环化改造、工业废弃物综合利用，提高能源利用效率。加强机动车尾气污染治理，淘汰高污染车辆，推广新能源汽车，加快发展绿色交通。有序推进农村冬季取暖清洁能源改造。开展工业企业污染、矿山环境恢复治理，实施土壤污染治理与修复工程。着力建设节水型社会，落实河长制、湖长制，提升水环境质量。严格落实生态环境损害责任追究制。

提高规划建设品位。发挥规划引领和刚性管控作用，修订完善各类专项规划，全面推进“多规合一”，构建单体建筑特色鲜明，区域风格整体协调，产业布局、生态环境、历史文脉相融合的高水平规划体系。严格执行建设用地控制标准，强化土地供应全程监管，提高城市空间和土地利用效率。持续加强城市基础设施建设，实施地下管网、污水处理厂升级改造等项目。完成“三供一业”分离移交改造任务。继续实施棚户区改造项目，完成征迁改造房屋659套，改造老旧小区住房10060套。

加强城市精细管理。加快城市建设管理立法，运用法治化、社会化、智能化、标准化手段，下足绣花功夫精细管理城市。加大城市精准整治力度，清理占道经营、污水直排、占用盲道等行为，合理规划公交路线，推行道路泊车智能化、市场化管理。加快生活垃圾分类试点城市建设，实施餐厨垃圾统一处理、生活垃圾分类收集、建筑垃圾回收综合利用等重点项目。加强再生资源回收网点整治和集中经营管理。落实“门前五包”责任制。深入开展全域全城无垃圾创建活动。建立物业管理联动机制，提高物业服务整体水平。推进以5G技术为引领的新型智慧城市建设。推动新一轮全国文明城市创建，持续提升市民整体文明素质和城市文明程度。

（七）纵深推进改革开放，切实增强发展活力。坚持用改革的思维破解难题，用开放的办法拓展发展空间，为高质量发展注入新活力、增添新动力。

深化国资国企改革。加快资源资产整合重组，继续做好企业公司化改制，建立公司法人治理结构，推进资源资产化、资产资本化、资本证券化。积极发展混合所有制经济，选择具备条件的国有企业开展员工持股试点，鼓励民营企业参与国有企业混合所有制改革。加快嘉恒公司、文旅集团等改制企业整合重组进度，完成大友公司等企业公司化改制。实施工资总额预算管理，加强市属企业负责人员经营业绩考核。加大国资监管力度，促进国有资产提质增效，力争国有资本经营收益突破3000万元。

抓好重点领域改革。推进投融资体制改革，充分利用资产证券化、产业基金、国有资源置换等办法，扩充基础设施建设项目资本金。落实财税体制改革任务，完善政府举债融资机制，规范国企投融资行为，遏制债务增量，化解债务存量。实施预算绩效管理，推动财政资金聚力增效。强化市场对资源的配置，减少政府干预，加强事中事后监管，凡市场能自主调节的交给市场，凡企业能干成的交给企业。

提升对外开放水平。支持酒钢集团等重点企业走出去，开展国际产能合作。鼓励有条件的企业承接产业梯度转移，重点面向中西亚、中东欧市场开发生活日用、轻工五金等产品。建成霍尔果斯口岸办事处，积极开展对外贸易。开展与“一带一路”沿线国家和地区文化旅游推介活动，积极参加兰洽会、文博会、亚欧博览会等展会。加大嘉酒区域联合协作力度，拓展区域经济发展空间。重点在文化、教育、旅游、城市建设、社会治理等领域，加强与北京市西城区、西班牙潘普洛纳市等友好城市合作交流。

（八）持续优化营商环境，不断提升服务水平。深化“转变作风改善发展环境建设年”活动，解决群众办事难点、企业发展痛点、社会各界反映堵点，打造政策优、成本低、服务好、办事快的营商环境。

继续深化“放管服”改革。深入推进“马上办、网上办、就近办、一次办”改革，强化政管办职能，以便民快捷原则整合政务中心，全面实现“一窗受理，集成服务”。进一步压减办事流程和环节，投资项目全流程审批时间再压缩一半。整合共享政务数据资源，提升一体化在线政务服务平台保障水平，确保所有政务服务事项网上可办率90%以上。继续推进政务服务向基层延伸，扩大社区“就近办”事项，实现“最多跑一次”城乡全覆盖。推进跨部门“双随机、一公开”监管信息和社会信用信息共享，推动事前事后监管信息与政务服务深度融合。

深入开展“企业服务年”活动。健全政府与企业常态化沟通机制，靠实市级领导联系服务重点企业责任，定期召开酒钢集团、中核四〇四、中核龙瑞公司等地企联席会和政银企对接会、企业座谈会。落实促进民营经济健康发展意见“30条”及配套措施，帮助解决企业发展存在的实际困难。全面落实减税降费政策，扩大“零收费”范围，切实减轻企业负担。支持银行金融机构扩大授信规模，力争民营企业贷款融资占比达到20%以上。完善融资担保体系，市级财政向中小企业金融服务公司注资2000万元，有效破解民营企业融资难题。搭建产销对接合作平台，支持企业抱团发展。整治政府部门、国有企业拖欠民营企业款项、“新官不理旧账”等行为，疏堵结合化解企业“三角债”。开展第三方评估，建立企业参与营商环境政策制定工作机制。加快构建亲清政商关系，对损害企业利益和营商环境的行为，坚决依法依规追责。弘扬企业家精神，为企业提供法律援助，保护民营企业家合法权益和人身财产安全。

（九）培育良好创新生态，创新驱动引领发展。深入实施创新驱动发展战略，着力构建多领域互动、多要素联动的创新生态体系，充分释放创新潜能，引领产业转型升级。

积极创建国家创新型城市。对标建设创新型城市指标体系，研究制定加快培育良好创新生态、建设国家创新型城市实施意见，全面启动创建工作。用足用好省级科技创新券、专利资助等优惠政策，优化整合各类资金，加大科技创新投入。围绕传统产业改造升级和战略性新兴产业发展，加快组建一批产业技术联盟和科技协同创新体，集中力量组织实施一批省、市级重大科技专项。

支持企业创新发展。突出企业创新主体地位，鼓励企业建设更多重点实验室和工程技术研发中心，加快省级高新技术产业开发区建设进程。实施“科技创新苗圃”“创业创新孵化”和“中小企业成长”计划，加快推进“创生个、个升限、个转企、小升规、规改股、股上市”，支持大中小企业、线上线下融通创新。深化科技金融改革创新，完善科技型企业“投、贷、债、保”联动机制。深入推动大众创业万众创新，加快创客空间、创新工场等新型孵化器建设，打造一批低成本、开放式众创空间。

加强人才队伍建设。修订完善深化人才发展体制机制改革若干意见“58条”，建立更具竞争力和吸引力的人才集聚制度。坚持直接引才和柔性引才相结合，统筹抓好创新创造科技领军人才、技术技能人才、青年优秀人才、企业家人才等各类人才培养。坚持“一人一策”，为“高精尖缺”人才在编制、住房、子女入学、配偶安置、医疗保障等方面开辟绿色通道。完善人才评价激励和收入分配制度，激发各类人才创新创业活力，让为经济社会发展作出贡献的人，得到应有经济报酬、政治待遇和社会荣誉。

（十）持续保障改善民生，不断增进人民福祉。坚持以人民为中心的发展思想，保障财政支出重点投入民生领域，让人民群众更多更好地共享改革发展成果。

加强就业和社会保障。落实就业创业扶持政策，实施高校毕业生就业创业计划和基层成长计划，引导鼓励大学生、农民工等返乡人员创业就业，全年新增就业8000人左右。开展失业率调查，城镇登记失业率控制在3%以内。推动“无欠薪”城市创建，解决好农民工工资拖欠问题。推进社会保险经办改革，提高社会保险待遇标准和服务水平。全面落实社会保障惠民政策，提高城乡低保、医保补助水平和特困人员救助供养补助标准，维护残疾人、老年人、留守妇女儿童等弱势群体利益，做好社会救助兜底保障工作。

统筹推进各项社会事业。持续加大教育投入，扩大优质学前教育资源供给，推进义务教育优质均衡发展，深化职业教育产教融合、校企合作改革，实现福民街、建林街、南市区幼儿园及小学、师范附校初中部开班招生，完成实验中学教学楼主体工程，开工建设南市区中学等项目。深化医药卫生体制改革，完善现代医院管理制度，完成中医院急诊楼维修改造，加快南市区医院投入使用。推进居家和社区养老服务改革试点，创建可复制可推广的嘉峪关模式。持续构建现代公共文化服务体系，出台更多文化惠民举措，打造群众性文化活动品牌；争创全民运动健身模范市，举办第二届全民健身健康大会，不断丰富群众文化体育生活。

加强和创新社会治理。深化平安嘉峪关建设，完善立体化社会治安防控体系，实施“雪亮工程”。强化网格化服务管理。深入开展“扫黑除恶”专项斗争。严厉打击电信网络诈骗、非法集资和非法金融活动，维护社会和谐稳定。认真做好信访工作，加大矛盾纠纷排查化解力度。落实安全生产责任，创建全国安全发展示范城市，强化重点领域安全监管和隐患排查治理，提升防灾减灾、应急救援、事故处置能力，保障人民群众生命财产安全。完成统一届期村委会（居委会）换届选举，确保民主参选率95%以上。做好退役军人管理服务和就业，维护军人军属合法权益。做好创建全国双拥模范城“七连冠”、全省双拥模范城“九连冠”验收评选工作。

继续办好惠民实事。突出普惠性、保基本、均等化、可持续原则，解决群众急难愿盼问题，办好提高城镇职工大病医疗保险保障水平、社区养老服务向农村延伸、酒钢职工游乐园环湖步道改造、老旧居民小区改造提升、农村居民庭院和生产道路改造、市残疾人康复中心建设6件惠民实事。

三、加强政府自身建设

面对新形势、新任务，我们将按照“信念过硬、政治过硬、责任过硬、能力过硬、作风过硬”的要求，加强自身建设，持续转作风、提效能，努力向全市人民交出满意答卷。

自觉提高政治力。坚持以习近平新时代中国特色社会主义思想和党的十九大精神为指引，始终把讲政治贯穿政府工作全过程，树牢“四个意识”，坚定“四个自信”，坚决做到“两个维护”，自觉在思想上政治上行动上同以习近平同志为核心的党中央保持高度一致。严守政治纪律和政治规矩，注重用政治眼光对待、分析和处理问题，强化政治责任，保持政治定力，提高政治能力，确保各项工作始终沿着正确方向前进。

全面提高谋划力。按照“干什么学什么、缺什么补什么”原则，加强对新理念、新知识、新方法的学习，进一步提升专业化能力，打牢全面系统的知识根基。坚持解放思想与实事求是相结合，增强战略思维、辩证思维、系统思维、底线思维，对项目建设、产业接续、财源培植、环境保护、民生改善等各领域工作深入谋划论证，做到上接“天线”、下接“地气”，既符合发展实际、又顺应群众意愿。

切实提高担当力。树立“功成不必在我、功成必定有我”的责任担当，对定下的目标、看准的事情扭住不放、一抓到底，全力以赴攻克制约发展的矛盾和难题。强化敢于担当、敢为人先的意识，把谋事、干事、成事、不出事统一起来，少讲不能办、多想如何办，创造性推动各项工作开展。全面落实鼓励激励、容错纠错机制，着力解决不担责怕担责、不作为慢作为等问题，推动政府系统广大干部轻装上阵、放开手脚干事创业。

坚决提高执行力。把抓落实作为政府工作的生命线，树立“埋头苦干、务实重行”的作风，坚持“俯身抬头”工作法，做到低调务实不张扬，集中精力抓落实。大兴调查研究之风，扑下身子、沉到一线，深入基层了解群众所思所盼、市场主体所想所急。坚持马上就办、干就干好，坚决纠正懒政怠政行为，提升政府执行力和公信力。落实好机构改革任务，确保工作前后衔接不断档、执行高效不误事。

持续提高约束力。严格落实全面从严治党责任，认真履行“一岗双责”，把政府系统党风廉政建设不断引向深入。严格执行中央八项规定及实施细则精神，下大气力解决“四风”问题，牢固树立“过紧日子”思想，严控一般性支出，把有限的资金用在刀刃上。坚持依法履行职责，推进依法行政和法治政府建设，落实公职律师、法律顾问、专家咨询等制度，自觉接受人大依法监督、政协民主监督、监察专责监督、司法监督和社会舆论监督，推动决策科学化、民主化、法治化。强化财政支出、工程建设、公共资源交易等重点领域监管，规范权力运行，堵塞风险漏洞，严肃查处违纪违法行为，切实以清明廉洁的政府形象赢得人民群众的支持和信任。

各位代表，新的一年开启新的希望，新的征程承载新的梦想。让我们更加紧密地团结在以习近平同志为核心的党中央周围，在省委、省政府和市委的坚强领导下，在市人大、市政协和社会各界的监督支持下，坚定信心、鼓足干劲，咬定青山不放松，撸起袖子加油干，加快推进高质量转型发展，实现高水平全面小康，以优异成绩迎接中华人民共和国成立70周年！

注释：

1.十大绿色生态产业：2018年1月，省委、省政府决定发展清洁生产、节能环保、清洁能源、先进制造、文化旅游、通道物流、循环农业、中医中药、数据信息、军民融合十大生态产业。

2.旅游人数和旅游收入：2018年全市旅游接待人数1045万人次，收入73.6亿元，分别增长22%和29%。旅游人数指离开居住地10公里以上、游览超过6小时的游客总和。统计数据为全年各旅游景区、星级饭店、乡村农家乐、民宿、旅行社、社会酒店接待总人次数之和（每单位接待1人统计为1人次）。旅游收入指一日游游客收入（旅游总人次数×60%×587.83元/人）+过夜游游客收入（旅游总人次数×40%×889.88元/人）之和。其中：60%和40%分别为一日游和过夜游游客比例；587.83元和889.88元为省文旅厅抽样调查确定一日游和过夜游的一天人均花费值。

3.“三变”改革：农村资源变资产、资金变股金、农民变股东改革。

4.“双促双增”：促进农业提质增效，促进农民持续增收，加快推动农村全面振兴。

5.“政府服务中心+分中心+社区服务中心”：市政府政务服务中心，社会保障、市场监管、税务、不动产登记、公安办证、车辆管理、住房公积金7个分中心，34个社区（镇）服务中心。

6.2018年省政府为民办实事涉及我市6件：农村危房改造、农村妇女“两癌”检查、助残扶贫康复项目、城市老旧住宅小区电梯改造、城镇棚户区改造、农村初中温暖工程。其中城镇棚户区改造项目正在推进，已完成1062套、开工改建12750套，其他5件实事全面完成。

7.2018年市政府8件惠民实事：开通三镇公交车、关城景区向市民永久免费开放、城乡公厕建设、全民健身设施建设改造、重点公共区域免费WiFi、“互联网+政务服务”、雄关便民卡、高铁南站电梯配置8件实事全面完成。

8.省政府“十不准”规定：不准触碰越过一条线、不准推脱分内一丝责、不准拖延超时一分钟、不准虚化落空一件事、不准虚报假报一个数、不准粗暴对待一个人、不准随意违悔一条约、不准劳民多跑一趟路、不准违规收受一份礼、不准干好干坏一个样。

9.中央“五坚持”大政方针：坚持稳中求进工作总基调，坚持新发展理念，坚持推进高质量发展，坚持以供给侧结构性改革为主线，坚持深化市场化改革、扩大高水平开放。

10.“六稳”工作：稳就业、稳金融、稳外贸、稳外资、稳投资、稳预期。

11.“僵尸企业”：已停产、半停产、连年亏损、资不抵债，主要靠政府补贴和银行续贷维持经营的企业。

12.一馆两中心：市档案馆、全民健身中心和后勤保障服务中心。

13.“大棚房”：以农业大棚为名进行非农业建设，属违法建设，主要包括三类：一是在各类农业园区内占用耕地或直接在耕地上违法违规建设非农设施，特别是别墅、休闲度假设施；二是在农业大棚内违法违规占用耕地建设商品住宅；三是建设农业大棚看护房严重超标准，甚至违法违规改变性质用途，进行住宅类经营性开发。

14.“门前五包”：包卫生、包秩序、包文明、包绿化、包安全。

15. 5G技术：第五代移动电话行动通信标准，也称第五代移动通信技术，缩写：5G，是4G之后的延伸。

16.“创生个、个升限、个转企、小升规、规改股、股上市”：“创生个”指支持农民、高校毕业生、退役军人、返乡创业人员、失业人员、科技人员等各类群体创业发展为个体工商户；“个升限”指个体工商户升级为限额以上个体工商户；“个转企”指个体工商户转型升级为小微企业；“小升规”指小微企业升级为“四上”企业（规模以上工业企业、资质等级建筑业企业、限额以上批零住餐企业、国家重点服务业企业）；“规改股”指“四上”企业改制为股份制企业；“股上市”指推动具备条件的企业上市（挂牌）。

17.“雪亮工程”：以综治中心为指挥平台、综治信息化为支撑、网格化管理为基础、公共安全视频监控联网应用为重点的“群众性治安防控工程”。

18.政治力：即政治能力，指政“德”，领导干部要有坚定的政治方向、政治立场、政治观点和政治鉴别力、政治敏锐性，学会用政治的眼光看待和分析问题，按照讲政治的要求处理问题。
